# The Hemorrhagic Coli Pilus (HCP) of *Escherichia coli* O157:H7 Is an Inducer of Proinflammatory Cytokine Secretion in Intestinal Epithelial Cells

**DOI:** 10.1371/journal.pone.0012127

**Published:** 2010-08-12

**Authors:** Maria A. Ledesma, Sara A. Ochoa, Ariadnna Cruz, Luz M. Rocha-Ramírez, Jaime Mas-Oliva, Carlos A. Eslava, Jorge A. Girón, Juan Xicohtencatl-Cortes

**Affiliations:** 1 Laboratorio de Bacteriología Intestinal, Departamento de Infectología, Hospital Infantil de México Federico Gómez, México Distrito Federal, México; 2 Instituto de Fisiología Celular, Universidad Nacional Autónoma de México, México Distrito Federal, México; 3 Departamento de Salud Pública, Facultad de Medicina, Universidad Nacional Autónoma de México, México Distrito Federal, México; 4 Department of Molecular Genetics and Microbiology, Emerging Pathogens Institute, University of Florida, Gainesville, Florida, United States of America; Columbia University, United States of America

## Abstract

**Background:**

Enterohemorrhagic *Escherichia coli* (EHEC) O157:H7, the causative agent of hemorrhagic colitis and the hemolytic uremic syndrome (HUS), produces long bundles of type IV pili (TFP) called hemorrhagic coli pili (HCP). HCP are capable of mediating several phenomena associated with pathogenicity: i) adherence to human and bovine epithelial cells; ii) invasion of epithelial cells; iii) hemagglutination of rabbit erythrocytes; iv) biofilm formation; v) twitching motility; and vi) specific binding to laminin and fibronectin. HCP are composed of a 19 kDa pilin subunit (HcpA) encoded by the *hcpA* chromosomal gene (called prepilin peptidase-dependent gene [*ppdD*] in *E. coli* K-12).

**Methodology/Principal Findings:**

In this study we investigated the potential role of HCP of *E. coli* O157:H7 strain EDL933 in activating the release of pro- and anti-inflammatory cytokines from a variety of host epithelial cells. We found that purified HCP and a recombinant HcpA protein induced significant release of IL-8 and TNF-α, from cultured polarized intestinal cells (T84 and HT-29 cells) and non-intestinal HeLa cells. Levels of proinflammatory IL-8 and TNF-α, but not IL-2, IL6, or IL-10 cytokines, were increased in the presence of HCP and recombinant HcpA after 6 h of incubation with ≥50 ng/ml of protein, suggesting that stimulation of IL-8 and TNF-α are dose and time-dependent. In addition, we also demonstrated that flagella are potent inducers of cytokine production. Furthermore, MAPK activation kinetics studies showed that EHEC induces p38 phosphorylation under HCP-producing conditions, and ERK1/2 and JNK activation was detectable after 3 h of EHEC infection. HT-29 cells were stimulated with epidermal growth factor stimulation of HT-29 cells for 30 min leading to activation of three MAPKs.

**Conclusions/Significance:**

The HcpA pilin monomer of the HCP produced by EHEC O157:H7 is a potent inducer of IL-8 and TNF-α release, an event which could play a significant role in the pathogenesis of hemorrhagic colitis caused by this pathogen.

## Introduction

Enterohemorrhagic *Escherichia coli* O157:H7 (EHEC) causes infection that range from asymptomatic or mild diarrhea to hemorrhagic colitis, in some cases causing Hemolytic Uremic Syndrome (HUS) that may lead to death [1]–[3]. Several factors contribute to the virulence of EHEC. Shiga toxins (Stx), also known as verocytotoxins damage the kidney, renal endothelial cells and block the microvasculature by toxicity and induction of local cytokine and chemokine production which leads to renal inflammation [4].

Interleukin-8 (IL-8) is one of the most important chemokines and chemoattractants that recruits neutrophils to the site of infection. Previous studies have shown that some enteropathogens induce target epithelial cells to produce this cytokine causing only mild gastroenteritis [5], [6]. *Shigella* infection triggers the production of IL-8 that can lead to epithelial cell destruction and histopathologic lesions of the colon [7].

Flagella of many bacterial pathogens are capable of activating the production of proinflammatory molecules in epithelial, monocytic, polymorphonuclear, and dendritic cells [8]. Activation of cytokines such as IL-8, IL-1β, tumor necrosis factor-α (TNF-α) and IL-6 is triggered by the recognition of flagellin monomers of many bacterial pathogens via Toll-like receptor 5 (TLR5) (for a review, see Ref. [9]. For example, the flagellins of EHEC O157:H7, enteroaggregative and enteropathogenic *E. coli* strains stimulate the secretion of IL-8 in target cells [10], [11]. TNF-α may be important in producing the pathologic changes observed in HUS and together with IL-8 could exhibit synergistic cytotoxic activity toward human endothelial cells [12]. Gewirtz *et al* showed that the flagella of *Salmonella enterica* serovar Typhimurium triggers basolateral IL-8 secretion from cultured model epithelia via Ca^++^-mediated activation of the NF-κB pathway [13]. They hypothesized that *S.* Typhimurium might trigger epithelial exocytosis of a proinflammatory mediator that could activate IL-8 synthesis via a mechanism similar to TNF-α [14]. Xicohtencatl *et al* [8] showed that purified flagella from *Vibrio cholera*e and other flagellins (FlaA, FlaC, and FlaD) found in cell-free supernatants are involved in the induction of IL-8 in epithelial cells. The two other flagellin subunits FlaB or FlaE, could not be detected in the supernatants studied; nevertheless, it is possible that they might be secreted in the gut environment and have IL-8 induction capability.

IL-8 gene expression is regulated by several pathways and their promoter region contains binding sequences for diverse transcription factors including IL-6, NF-κB and AP-1 [15]. NF-κB is a central regulator of the epithelial-cell innate immune response to infection by enteroinvasive bacteria [16]. AP-1 activation is dependent on mitogen-activated protein kinases (MAPKs), which form a group of three signaling pathways, including those for extracellular signal-regulated protein kinases (ERK1/2) and two stress-activated protein kinases named p38 and c-Jun N-terminal kinase (JNK). EHEC O157:H7 is capable of activating expression of p38 and ERK MAP kinases and the nuclear translocation of the transcription factor NF-κB [17].

Type IV pili (TFP) are important virulence factors in many pathogenic Gram-negative bacteria that can mediate adherence to eukaryotic cells, twitching motility, biofilm formation, DNA binding and bacterial aggregation [18]–[24]. The genome of *E. coli* O157:H7 contains several loci coding for fimbriae whose function in *in vivo* infections remains largely unknown for most of them [25]–[27]. Recently, the production of TFP called HCP (**h**emorrhagic **c**oli **p**ilus) was reported in EHEC. HCP was shown to be involved in adherence to epithelial cells and to porcine and bovine intestinal explants [24], invasion, hemmaglutination, biofilm formation, twitching motility, and extracellular matrix glycoprotein binding [28].

The role of HCP in the activation of proinflammatory molecule expression in epithelial cells has not been explored. In this study we investigated the role of HCP produced by EHEC O157:H7 in the activation and release of several proinflammatory and anti-inflammatory cytokines from human colonic epithelial cells (T84 and HT-29) and non-intestinal HeLa cells. These cells have been used in the past to study the ability of bacterial products to induce IL-8 and TNF-α activation [8], [10], [29], [30]. In addition, we evaluated the activation of MAPK (p38, ERK1/2) and NF-κB signaling pathways required for the induction of proinflammatory responses in intestinal epithelial cells. Lastly, we also assessed the role of flagella as inducer of cytokine production, particularly IL-8 and TNF-α by epithelial cells during EHEC infection.

## Results

### Production of HCP by EHEC O157:H7 strain EDL933

**Figure 1:**
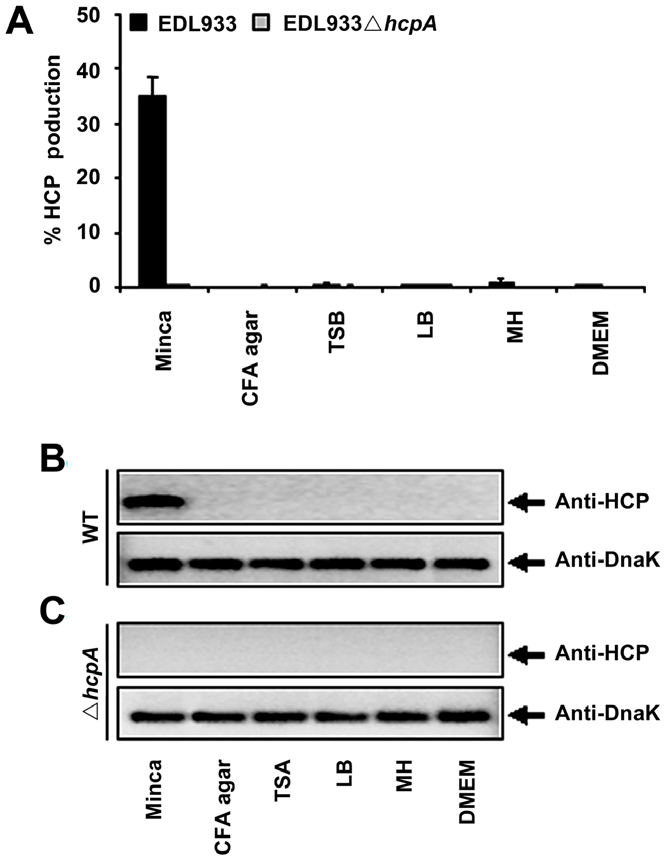
Production of HCP from EHEC EDL933 strain. Flow cytometry data from the EHEC EDL933 strain cultured in different media showing optimal production of HCP after bacterial growth in Minca (A). Western blot confirming HCP production by EDL933 in Minca (B). The EDL933Δ*hcpA* strain was used as a negative control (C). Detection of DnaK with anti-DnaK antibody was used to ensure that equal amounts of antigen were tested.

To evaluate the production of HCP from EHEC O157:H7, the EDL933 strain was cultured in CFA agar, Trypticase soy broth (TSB), Luria Bertani (LB), Mueller Hinton (MH), Dulbecco's Minimal Eagle Medium (DMEM) and Minca minimal medium (Fig. 1A). Our flow cytometry data indicated that 35% of the EDL933 bacteria grown in Minca were positive for HCP using anti-HCP (Fig. 1A). The presence of these pili was not detected when EDL933 was cultured in the other media (Fig. 1A) [24]. In addition, analysis by Western blot showed that EDL933 produced HcpA only in Minca and not in the other culture media tested, confirming the results obtained by flow cytometry (Fig. 1B). The EDL933 *hcpA* mutant strain was used as a negative control and did not cross react with anti-HCP antibodies, which confirmed the loss of the HcpA protein in this strain (Fig. 1C). To verify that equal amounts of antigen tested in the Western blot, anti-DnaK antibodies were used to detect the DnaK protein (Fig. 1B and C).

### Immunogold and Purification of HCP and His-tagged HcpA

**Figure 2:**
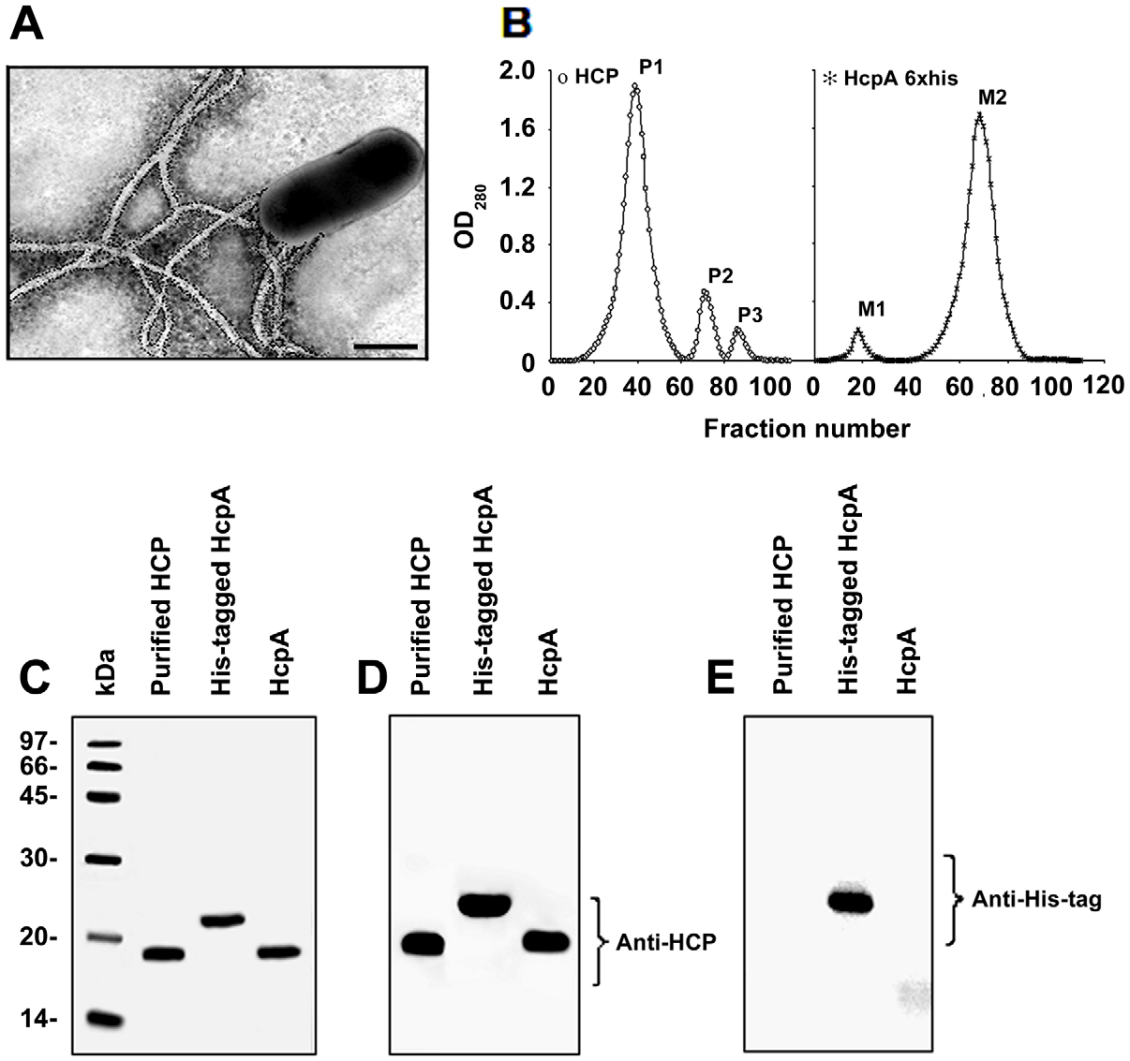
Immunodetection of HCP and the His-tagged HcpA protein. Immunogold-labeling of HCP produced by EDL933 grown on Minca agar using anti-HCP antibody (A). Scale bars, 0.5 µm. HCP and the HcpA protein (without the His-tag) were purified separately using a molecular exclusion chromatography (B). Purified HCP and the recombinant protein were depolymerized in 16% SDS-PAGE gels and stained with Coomassie blue (C). Peak P1, is the highest protein peak that showed a protein of 19 kDa corresponding to the pilus subunit HcpA. Peak M2 obtained during HcpA-His purification showed a protein that migrated as a 22 kDa protein due to the presence of 6 histidines. Western blot of both proteins with anti-HCP demonstrated the presence of the pilin (D). Western blot of HcpA His-tagged protein, showing a band of 22 kDa detected with monoclonal anti-His tag antibodies (E).

Immunoelectron microscopy studies of EDL933 grown in Minca agar revealed the presence of polar fimbrial structures, which were recognized by anti-HCP antibodies, confirming the identity of HCP (Fig. 2A). No reactivity was seen with rabbit pre-immune serum (data not shown). HCP produced by EDL933 were obtained after shearing and differential centrifugation, applied onto a molecular exclusion chromatography column and 0.5-ml fractions were collected (Fig. 2B). Protein peaks were analyzed by sodium dodecyl sulfate polyacrylamide gel electrophoresis (SDS-PAGE) gels stained with Coomassie blue and by Western blotting using anti-HCP and anti-His-tag antibodies. In the first peak (P1), as expected, we detected the presence of a protein band with a molecular weight of 19 kDa, which was identified as HcpA (Fig. 2B and 2C). Protein peak 2 (P2) and protein peak 3 (P3) showed the presence of two proteins of 30 and 14 kDa, respectively (Fig. 2B and data not shown). The identity of these three protein bands was assessed using specific antibodies against HCP. Western blotting experiments showed that the 19-kDa protein found in P1 reacted with anti-HCP antibodies, confirming the presence of the HcpA structural subunit of HCP (Fig. 2D). Proteins in peaks P2 and P3, did not react with anti-HCP antibodies indicating that these proteins are not related to HCP (data not shown).

The *hcpA* gene of EDL933 was cloned by PCR and the HcpA protein was expressed as a fusion protein containing a 6×His-tag at the N-terminus. The His-HcpA protein fusion was purified under native conditions using imidazole buffer in gradient concentrations of 100 to 250 mM. The fractions collected showed two protein peaks (M1 and M2) (Fig. 2B) which showed two bands of 66 and 22 kDa in 16% SDS-PAGE gels. Western blot analysis showed that the 22-kDa protein reacted with anti-HCP and anti-6×His-tag monoclonal antibodies whereas the 66-kDa protein did not react with anti-HCP antibodies (Fig. 2C, D and E and data not shown). Cleavage of the 6×His-tag of the HcpA protein yielded a 19-kDa protein reactive with anti-HCP antibodies (Fig. 2C, and D). No reaction was observed when this protein was incubated with anti-6×His-tag monoclonal antibodies (Fig. 2E).

### HCP Binds to Polarized HT-29 Cells

**Figure 3:**
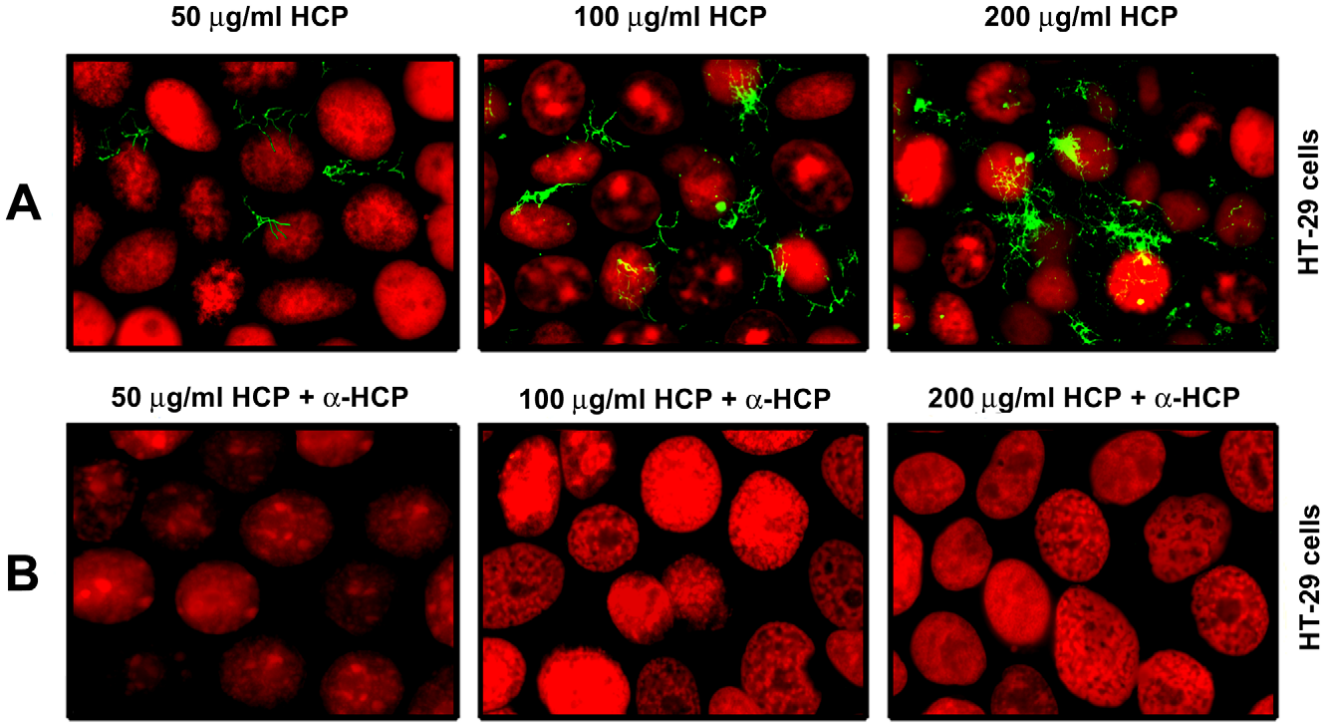
Binding properties of HCP to HT-29 cells. HCP used at 50, 100, and 200 µg/ml were incubated with polarized HT-29 cells, washed, and then visualized by IFM (A). Inhibition of HCP binding using a 1∶100 dilution of anti-HCP antibodies (B). The nucleus was labeled with propidium iodide (red) and HCP with Alexa Flour 488 (green). The images were taken at 200×.

Studies *in vitro* indicate that HCP from EHEC O157:H7 mediates adherence to human (intestinal and non-intestinal) and bovine epithelial cells [24]. We sought to demonstrate direct binding of purified HCP to the apical surface of HT-29 cells. Purified HCP used at concentrations of 50, 100, and 200 µg/ml were incubated with cultured polarized HT-29 cell monolayers and after removal of unbound pili, HCP bound to the apical surface of the cells were detected by IFM (immunofluorescence microscopy) using anti-HCP antibodies (Fig. 3A). Dose-dependent binding of HCP interacting with HT-29 cells was observed showing an increase of this pilus attached to the surface of the mammalian cells at high concentrations of protein (Fig. 3A). In support of these findings, when HCP was pre-incubated for 2 h with anti-HCP (1∶100) antibodies, the interaction of HCP with HT-29 cells was completely blocked (Fig. 3B). Preimmune serum used as negative control did not block the interaction of HCP with HT-29 cells (data not shown). These data suggest that HCP binds directly to epithelial cells through the recognition of a cell surface-exposed receptor.

### HCP Induced the Activation of Proinflammatory Cytokines in Polarized HT-29 Cells

**Figure 4:**
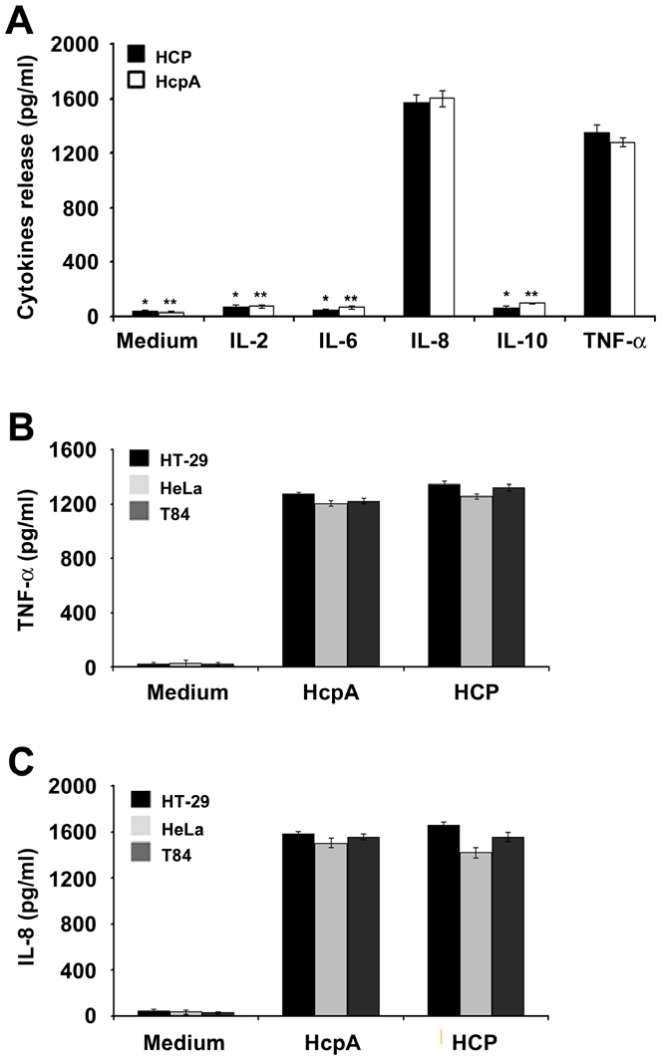
HCP and HcpA selectively activate release of IL-8 and TNF-α. HCP and HcpA induced release of IL-8 and TNF-α but not IL-2, IL-6, and IL-10 (A). *P<0.0001 and HcpA **P<0.001. High levels of IL-8 and TNF-α released from HT-29, T84, and HeLa cells in the presence of HCP and HcpA (B) and (C). HCP and HcpA induced similar levels of IL-8 (1658.3 pg/ml from HT-29; 1558.33 pg/ml from T84; and 1423 pg/ml from HeLa cells) (C). Both proteins induced comparable levels of TNF-α release from HT-29, T84, and HeLa cells [HCP (1346.6, 1258.3, and 1205.3 pg/ml) and HcpA (1272.6, 1205.3, and 1223.3 pg/ml)], respectively (B). Polarized intestinal and non-intestinal cells were incubated alone with DMEM as a negative control.

Several studies indicate that LPS, flagella, and pili of certain bacterial pathogens are potent activators of the secretion of a variety of proinflammatory cytokines [8], [10], [31], [32]. EHEC H7 flagellin, but not the Stx, is the major EHEC factor that directly upregulates proinflammatory chemokine production by human colon epithelium *in vivo* [33]. We investigated the potential role of HCP as inducer of proinflammatory (IL-2, IL-6, IL-8 and TNF-α) or anti-inflammatory (IL-10) cytokines from HT-29 human colonic epithelial cells. Polarized HT-29 cells were incubated with HCP and HcpA at the apical surface. Analyses of supernatants collected from the basolateral surface of HT-29 cells indicated that HCP and HcpA do not trigger IL-2, IL-6, and IL-10 release while significant high levels of IL-8 and TNF-α were observed (Fig. 4A).

However, when cytokines release at apical surface were tested, only IL-8 (15% induction) and TNF-α (20% induction) were detected at the basolateral surface; while IL-2, IL-6, and IL-10 were not detected in the apical surface (data not shown). After a comparative analysis of the results, we observed that HCP poorly induced the release of IL-2, IL-6 and IL-10 in comparison to TNF-α release (1354.33 pg/ml) (*P*<0.0001) (Fig. 4A). Likewise, similar results for HcpA were observed when the level of activation of TNF-α was compared to that of the latter interleukins (Fig. 4A). The levels of IL-8 and TNF-α release from polarized HT-29 cells were similar for HCP and the HcpA protein (without the 6×His-tag). When HcpA was analyzed, similar significant differences in the release of cytokines among IL-8 with the other interleukins (*P*<0.001) were obtained (Fig. 4A). In sum, cell-free supernatants obtained from the basolateral surface of HT-29 cells incubated with HCP for 6 h, showed low levels of IL-2 (73.66 pg/ml), IL-6 (44.33 pg/ml) and IL-10 (63.66 pg/ml) release compared to IL-8 (1570 pg/ml) release from polarized HT-29 cells (*P*<0.0001) (Fig. 4A). The results obtained with HcpA were practically identical to those obtained with HCP (*P*<0.001) (Fig. 4A). We also found that high levels of IL-8 and TNF-α induction were obtained upon incubation of HT-29 cells with pre-heated pili (monomeric pilin) and native pili (data not shown). All together, these are compelling data that support a role for HcpA as a potent inducer of proinflammatory cytokines.

### Cytokines Release in Intestinal and Non-Intestinal Cells

The secretion of IL-8 and TNF-α cytokines was monitored after 6-h incubation of different human polarized colonic (T84 and HT-29) and non-polarized non-intestinal (HeLa) epithelial cells with HCP and HcpA. We found that both forms of the proteins activate the release of IL-8 and TNF-α from the basolateral surface of these cell lines. The IL-8 release values were slightly higher than those for TNF-α release (Fig. 4B and C). For example, the levels for TNF-α for the different cell lines were: 1346.6±32.53 (HT-29), 1258.33±38.18 (T84), and 1205.3±30 pg/ml (HeLa), respectively (Fig. 4B). These values were significantly higher than those of the negative control (Fig. 4B). HT-29, T84, and HeLa cells incubated with HcpA showed TNF-α release values of 1272.6±20.40, 1205.3±17.89, and 1223.3±25.16, respectively (Fig. 4B).

As shown in Fig. 4C, both HCP and HcpA induced similar levels of IL-8 release form the 3 cell lines studied. In all, these data strongly suggest that HcpA and HCP trigger IL-8 release from the basolateral surface of these host epithelial cell lines and that the monomeric pilin subunit alone, is responsible for the proinflammatory activity of HCP displayed in host polarized and non-polarized epithelial cells. Purified flagella from EPEC and EHEC were used as positive controls to evaluate the release of IL-8 in intestinal cells (HT-29 and T84 cells) (data not shown). The IL-8 and TNF-α release values obtained in supernatants collected from the apical surface HCP and HcpA-treated HT-29 cells were significantly lower (5-fold reduction for IL-8 and 6.6-fold reduction) for TNF-α compared to those obtained from the basolateral surface (data not shown).

### Concentration and Time-Dependent Induction of IL-8 and TNF-α

**Figure 5:**
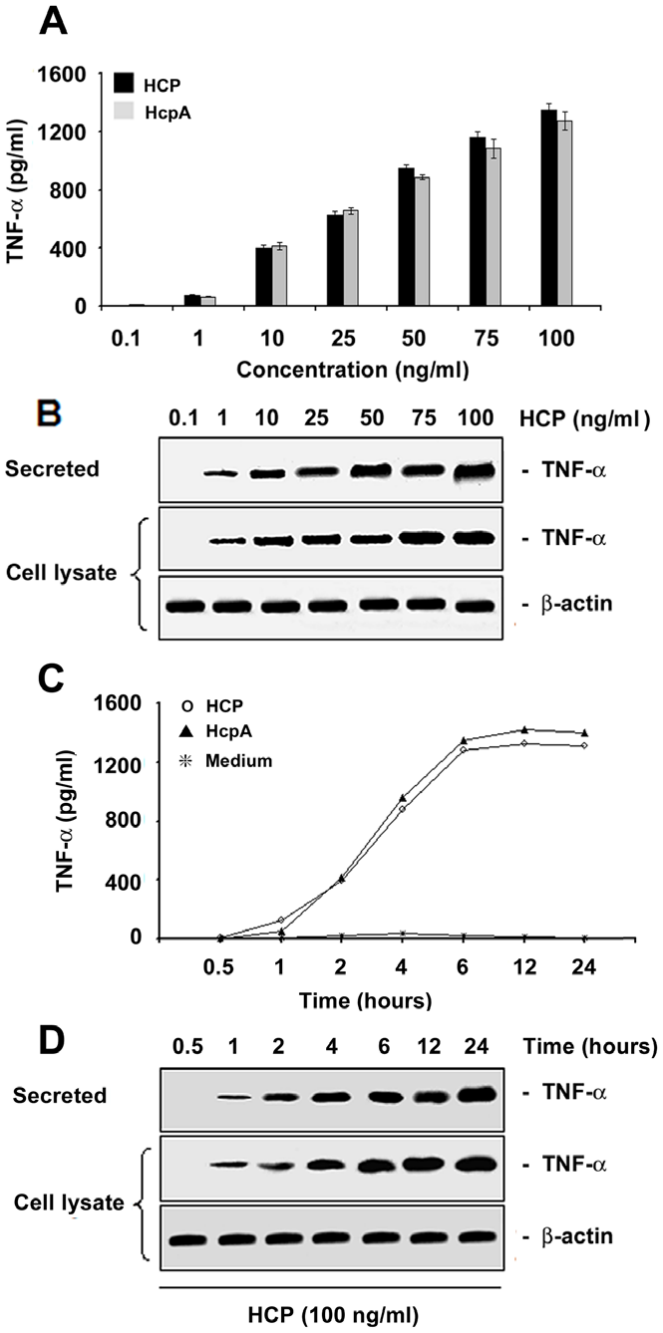
Induction of TNF-α release by purified HCP. An ELISA-based assay demonstrating the induction of TNF-α in polarized HT-29 cells in response to different concentrations of purified HCP and HcpA (A). Western blot showing the kinetics of secreted proteins and cell lysates produced after the incubation of polarized HT-29 cells with HCP and HcpA (B). Both proteins stimulate the production of TNF-α in HT-29 cells in a dose-dependent manner. Purified HCP (100 pg/ml) and HcpA (100 pg/ml) were incubated with polarized HT-29 cells at different intervals (C). The supernatant recovered from the basolateral surface showed a significant time-dependent increase of TNF-α as shown by Western blotting (D). Detection of actin was used as a protein loading control.

**Figure 6:**
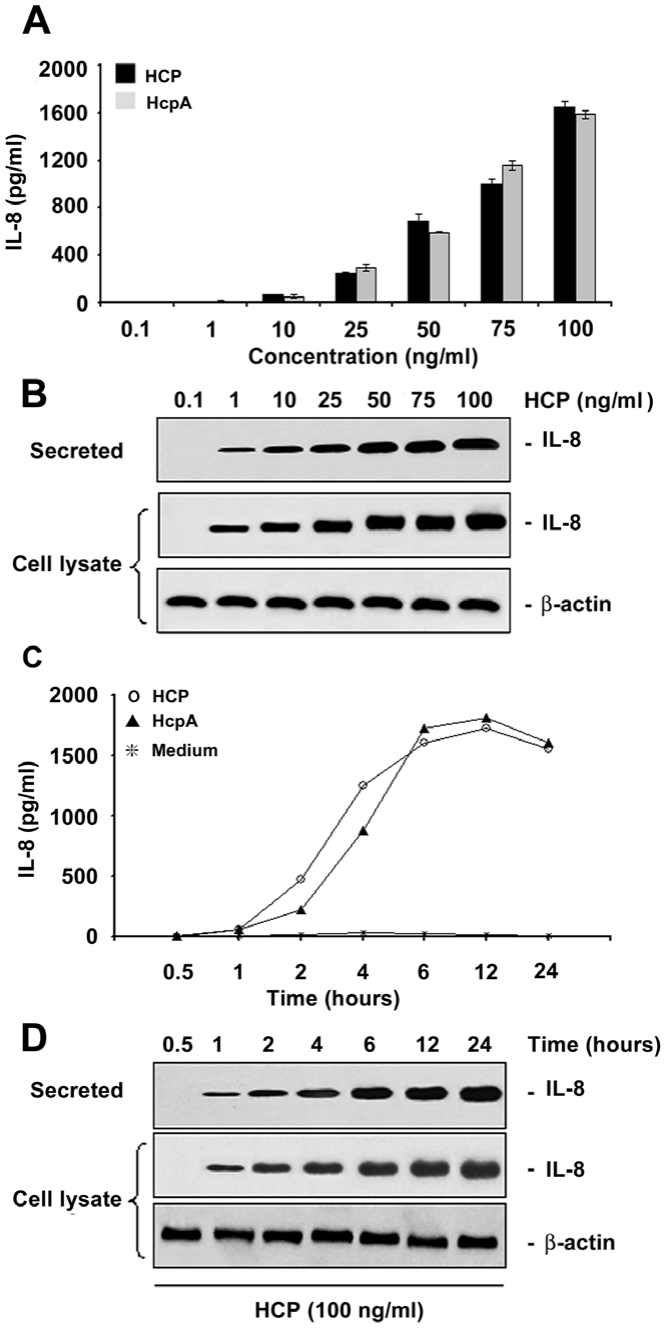
Induction of IL-8 release by purified HCP. IL-8 released in response to different concentrations of purified HCP and HcpA (A). Western blot showing the kinetics of secreted proteins and cell lysates produced after the incubation of polarized HT-29 cells with HCP and HcpA (B). IL-8 release after 6 h incubation of HCP with HT-29 cells (C). The supernatant recovered from the basolateral surface showed a significant time-dependent increase of IL-8 as shown by Western blotting (D). Detection of actin was used as a protein loading control.

HCP and purified HcpA were equally capable of inducing TNF-α and IL-8 release from HT-29 polarized human colonic cells in a time and concentration-dependent manner (Fig. 5 and 6). They induced fast and clear increases in TNF-α release of approximately ∼400 pg/ml at a concentration of 10 ng/ml and reached a maximum value of ∼1400 pg/ml at a concentration of 100 ng/ml of protein (Fig. 5A). The amount of TNF-α released was a function of the concentration of HCP (Fig. 5B). Detectable levels of TNF-α were seen after 2 h of incubation and a plateau was reached at 6 h (Fig. 5). Levels of TNF-α in cell lysates and supernatants were confirmed by Western blotting with anti-TNF-α antibodies (Fig. 5B and D). Detection of actin in whole cell extracts with monoclonal anti-actin antibodies was performed to ensure that equal amounts of antigen were tested at the different concentrations and time points. Identical TNF-α release values were obtained when T84 and HeLa cells were employed (data not shown).

In parallel, IL-8 induction by HCP and HcpA was tested at different concentrations of these proteins and during several incubation-time periods. As for TNF-α, no difference in IL-8 induction was seen between HCP and HcpA. However, IL-8 was detected only when 25 ng/ml of these proteins were assayed. These IL-8 detectable levels were obvious starting at 2 h of incubation reaching a peak at 6 h (Fig. 6A and C). Western blot analyses of cell lysates and supernatants confirmed the ELISA results (Fig. 6B and D).

### Antibodies Against HCP Blocked Proinflammatory Cytokines Release

**Figure 7:**
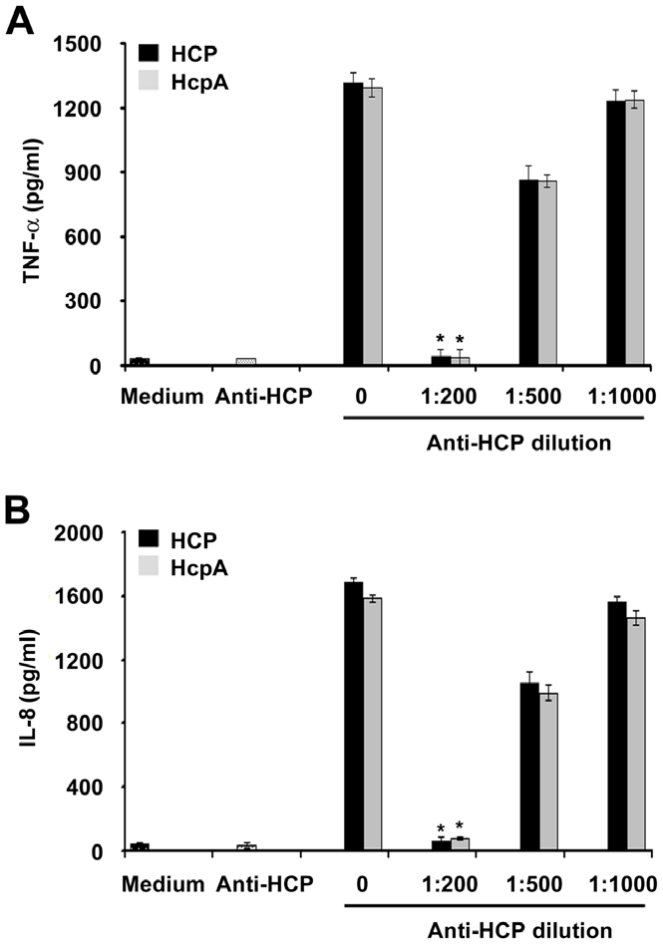
Anti-HCP antibodies blocked the secretion of proinflammatory cytokines. Purified HCP or HcpA protein (100 pg/ml) were pre-incubated with anti-HCP antibodies in DMEM low glucose medium before addition to the apical surface of HT-29 cells. A dose-dependent inhibition for IL-8 and TNF-α from HT-29 cells was obtained: between 95–98% at the 1∶200 dilution of the antibody (A and B). *P<0.0002.

To confirm that HCP induce proinflammatory cytokines (IL-8 and TNF-α) production in intestinal cells, these pili were pre-incubated for 2 h with anti-HCP antibodies (dilutions 1∶200, 1∶500 and 1∶1,000). We observed a dose-dependent inhibition of IL-8 and TNF-α secretion from HT-29 cells after 6 h of incubation with the antibodies (Fig. 7A and B). Maximal inhibition (∼98%) was obtained at the 1∶200 dilution of the antibody (*P*<0.0002) (Fig. 7A and B). When diluted 1∶500, a 33–37% reduction for TNF-α and 36–38% for IL-8 release was observed with both proteins (Fig. 7A and B). When the dilution was increased to 1∶1,000, we observed a poor reduction in TNF-α (7–10%) and IL-8 (6–8%) release (Fig. 7A and 7B); these three dilutions were compared with the release of the cytokines without the antibody. The preimmune serum used as the negative control was not able to block the IL-8 and TNF-α activation mediated by HCP and HcpA.

### EHEC Infection Increases IL-8 and TNF-α Release in HT-29 Infected Cells

**Figure 8:**
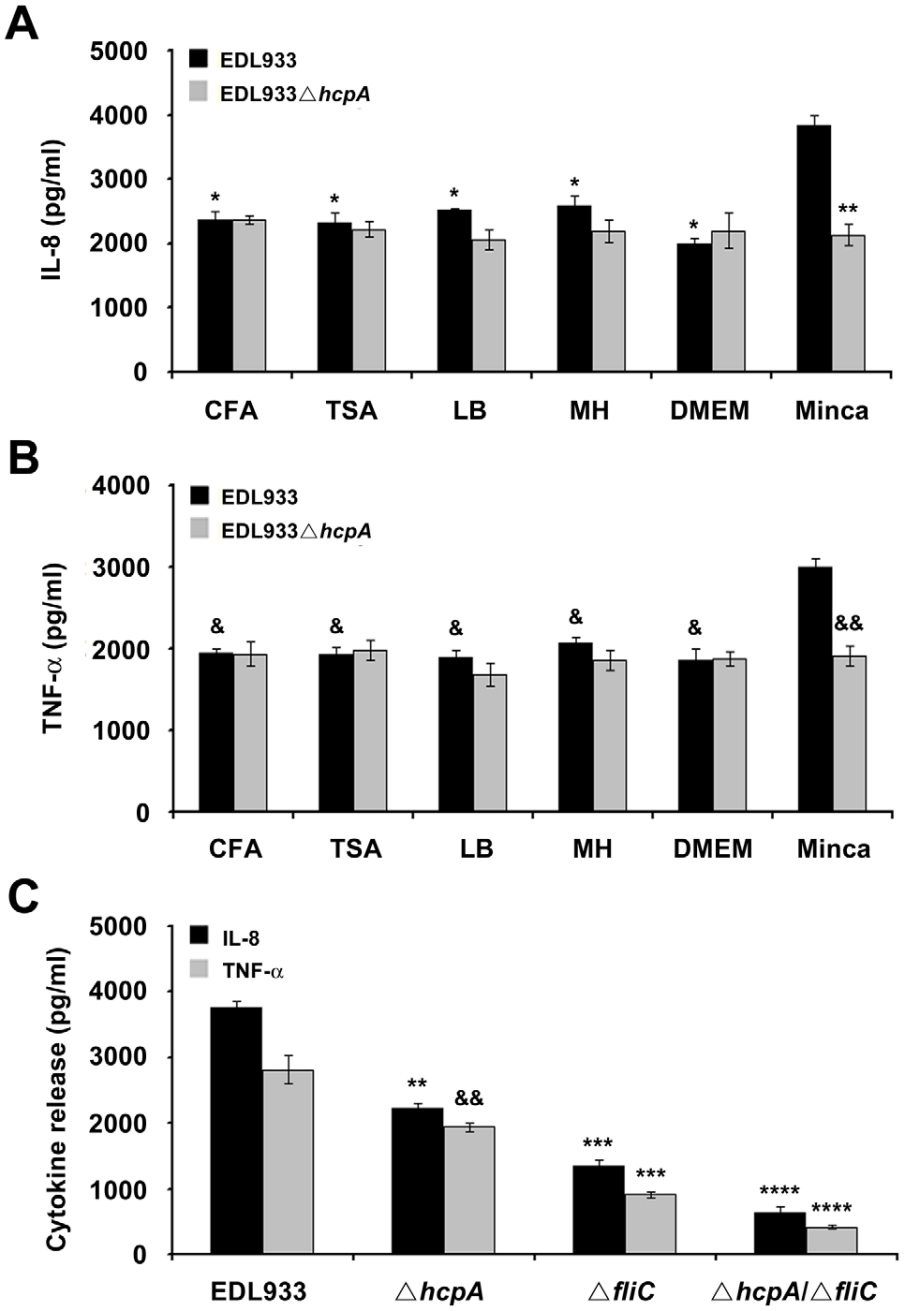
Comparison of the IL-8 release induced by EDL933 and derivative mutants after growth in different media. The highest level of IL-8 and TNF-α release was observed after growth of EDL933 in Minca (A and B) (*P<0.0001). The *hcpA* mutant was reduced for IL-8 and TNF-α induction compared to the wild-type strain (**P<0.0012). Comparison of IL-8 and TNF-α induction by EDL933, EDL933Δ*hcpA*, EDL933Δ*fliC* and EDL933Δ*hcpA*/Δ*fliC* previously cultured in Minca (C). Asterisks and ampersands indicate that a value is significantly different from the value for the mutant in *hcpA*, *fliC* and the double mutant *hcpA*/*fliC* cultured in Minca, **P<0.0012, ^&&^ *P*<0.005, ***P<0.001, and **** *P*<0.0001.

We tested if EHEC infection induces a pro-inflammatory response from epithelial cells measuring secretion of IL-8 and TNF-α in conditions where HCP is produced. EHEC strains were cultured in Minca to stimulate the activation and production of HCP and other media in which these pili are not produced. In addition, HT-29 cells were incubated with wild-type EDL933 or the *ΔhcpA* at the apical surface for 6 h. Although IL-8 and TNF-α levels were the highest when the bacteria were cultured in Minca, we also detected these cytokines in supernatants of cells infected with the wild-type and the *hcpA* mutant pre-grown in CFA, TSA, LB, MH, and DMEM (data not shown). In all, these data suggest that other bacterial surface components, in addition to HCP are present under these growth conditions that promote cytokine production (Fig. 8A and B). To provide an explanation for the residual activity in the *hcpA* mutant we used a flagella mutant of EDL933. When the *ΔfliC*, and *ΔhcpA/ΔfliC* mutants were analyzed and compared to the wild-type strain, it was apparent that both IL-8 and TNF-α levels were mostly reduced in the *hcpA/fliC* mutant followed by the *fliC* and then the *hcpA* mutant (Fig 8C). These results demonstrated that both flagella and HCP are potent inducers of IL-8 and TNF-α proinflammatory cytokines.

### HCP Activate NF-κB and AP-1 in HT-29 Intestinal Epithelial Cells

**Figure 9:**
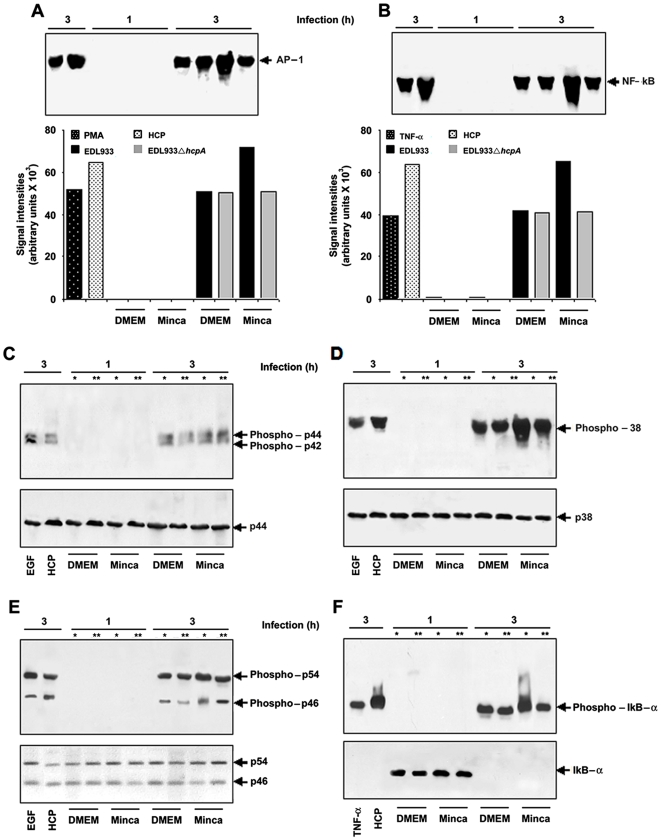
EHEC infection induced AP-1, NF-κB DNA binding, MAPK and IκB-α activation in HT-29 cells. Infection with purified HCP, wild-type O157:H7 and the *hcpA* mutant showing percentages of AP-1 activation; PMA: Phorbol myristate acetate (A). Infection with purified HCP, wild-type O157:H7 and the *hcpA* mutant showing percentages of NF-κB activation (B). Western blot showing the kinetics of MAPK activation using specific antibodies that recognize the phosphorylated forms of p42 and p44 (C). Wild-type EHEC is involved in the p38 phosphorylation in conditions where HCP is produced and a significant decrease was observed when tested with the EDL933 *hcpA* mutant (D). ERK1/2 and JNK activation were detectable after 3 h of EHEC infection (E). IκB-α activation was detectable after 3 h of EHEC infection (F). * EDL933 O157:H7 and ** EDL933Δ*hcpA*.

NF-κB and AP-1 play a central role regulating the activation of several proinflammatory cytokines such as IL-8, in human intestinal cells [15], [16], [34], [35]. We performed an Electrophoretic Mobility Shift Assay (EMSA) to investigate the ability of HCP produced by wild-type EDL933 and its derivative Δ*hcpA* mutant to activate NF-κB and/or AP-1 in polarized HT-29 cells when incubated for 1 and 3 h. Clear differences were noted at these two incubation periods. HCP and Minca-grown EDL933 bacteria showed different percentages of NF-κB and AP-1 activation after 3 h of incubation (Fig. 9A and B). Uninfected cells did not show NF-κB or AP-1 DNA binding activity (data not shown). We observed a significant increase of NF-κB and AP-1 activation with purified HCP and with the wild-type grown in Minca. In contrast, when the wild-type was cultured in DMEM it showed the same intensity of NF-κB and AP-1 DNA binding activation compared to the EDL933Δ*hcpA* cultured in DMEM or Minca (Fig. 9A and B). As a negative control a non-pathogenic *E. coli* DH5α strain was used, which showed a slight signal of NF-κB (data not shown). The analysis by densitometry showed that EHEC infection could lead to the activation of AP-1 DNA binding activity in similar percentages to those observed with NF-κB (Fig. 9A and B). The induction of NF-κB and AP-1 transcription factors is concomitantly activated after 3 h of infection of HT-29 cells with EDL933. Phorbol myristate acetate (PMA) involved in the transcriptional activation of AP-1 was used as a positive control (Fig. 9A). Translocation of NF-κB required the phosphorylation of IκB-α, which could be analyzed and detected by Western blot using specific antibodies (Fig. 9F). Phosphorylation of IκB-α was detected after 3 h of the infection and it was higher when the Minca-grown EDL933 was cultured in DMEM or the EDL933Δ*hcpA* strain cultured in DMEM or Minca (Fig. 9F).

### EHEC Activates MAPK in Human Intestinal Epithelial Cells

The mechanisms of the regulation of AP-1 activity involve aspects of phosphorylation by members of the MAPK family, including ERK1/2, p38, and JNK [36], [37]. We then determined if these signaling pathways (*e.g.* stimulation of MAPK modules) induce AP-1 activation upon EHEC infection of polarized HT-29 cells. Western blots were performed to evaluate the kinetics of MAPK activation using specific antibodies that recognize the phosphorylated forms of ERK1/2, p38 and JNK (Fig. 9C, D, and E). The active forms of ERK1/2 (p42 and p44), p38, and JNK (p46 and p54) were not detected in HT-29 cells with or without 1 h of incubation with TNF-α, purified HCP, EDL933 and the *hcpA* mutant (data not shown; Fig. 9C, D, and E). Our data showed that wild-type EHEC expressing HCP triggers hyper-phosphorylation of p38 while the EDL933Δ*hcpA* mutant does not (Fig. 9D). When DMEM-grown EDL933 and the *hcpA* mutant cultures were used in these experiments similar levels of p38 phosphorylation in HT-29 cells were observed. ERK1/2 and JNK activation was detectable only after 3 h of EHEC infection and when incubated with purified HCP (Fig. 9C and E). Likewise, HT-29 cells infected with EDL933 pre-cultured in Minca showed increased phosphorylation of p54 and p46, as compared to cells infected with EDL933 and the *hcpA* mutant harvested from culture conditions in which HCP is not produced (Fig. 9E). In the case of p44 and p42, no differences in the level of phosphorylation were observed, although HCP appears to induce their activation. As control of MAPK activation in these experiments, HT-29 cells stimulated with epidermal growth factor (EGF) for 30 min were used (Fig. 9C, D, and E).

## Discussion

The HCP of EHEC O157:H7 displays several biological functions associated with pathogenicity [24], however, not much is known regarding its immunobiological properties or the environmental and nutritional factors involved in the production of HCP. In many instances, *in vitro* growth conditions constitute signals that may typify or mimic certain biological niches. Environmental signals and molecular mechanisms are typically involved in regulation of virulence gene transcription, for example the Stx of EHEC, the toxin-coregulated pilus from *V. cholerae*, the bundle-forming pili (BFP) from EPEC, and the 987P pili of enterotoxigenic *E. coli* (ETEC) [38]–[42]. We previously reported production of HCP by EHEC O157:H7 after growth in Minca minimal media [24]. The fact that HCP is produced only in Minca and not in nutrient rich media or tissue culture medium is an indication that, like other *E. coli* fimbriae, expression of HCP genes is tightly regulated by nutritional signals. Minca medium has been used in the past to successfully induce expression of fimbriae in bovine ETEC [43]. It is possible that a yet unknown signal(s) is present in this medium that relieves the negative regulation of fimbrial genes exerted in other growth conditions. HCP are also produced upon contact of EHEC with cultured epithelial cells and explants of pig intestinal tissue suggesting that production of these pili is stimulated also by host signals [24].

Purified HCP bound specifically to cultured mammalian cells most likely via a yet unknown receptor and antibodies against HCP blocked this binding, supporting previous observations that HCP has adhesive properties [24]. The fact that HCP-producing strains are highly adherent to intestinal, non-intestinal and bovine cells suggests the presence of a common receptor in these cell lines [24].

Pathogen-associated molecular patterns (PAMPs) such as LPS, flagella, and pili are potent activators involved in the secretion of a variety of proinflammatory cytokines [8], [10], [31], [32]. Bacterial flagellins are potent inducers of proinflammatory molecules (IL-1β, IL-6, and IL-10) via TLR5 in epithelial cells [45], [46]. There is a growing interest in IL-8 and TNF-α as powerful neutrophil chemoattractants and activators. EHEC infections in humans are characterized by the presence of inflammation of the colon or hemorrhagic colitis [47]. *E. coli* O157:H7 strains produce virulence factors that contribute to the proinflammatory and immune responses of intestinal cells to this pathogen [14], [44]. Previous *in vivo* and *in vitro* EHEC infection studies demonstrated that the production of Stx induces an inflammatory response attributed to IL-1 and TNF-α [30], [44]. Thorpe *et al* [48] showed that verotoxins act as mediators of IL-8 secretion by intestinal cells. We sought to explore the contribution of HCP to the genesis of gut inflammation by measuring its potential as an inducer of proinflammatory cytokines in macrophages and epithelial cells (intestinal and non-intestinal) in response to infection. We found that host cells incubated with HCP or infected with EHEC O157:H7 are activated for the release of IL-8 and TNF-α, and phosphorylation of p38, ERK1/2 MAP kinases, and NF-κB. Several signaling pathways regulate the expression of the IL-8 gene [44]. In comparison to EPEC flagellin, which was previously shown to induce IL-8 release from T84 cells [10], HCP stimulated higher levels of IL-8 release from HT-29 cells. Dosage and kinetics studies showed that activation of IL-8 and TNF-α release dose and time-dependent. We also observed increased AP-1 DNA binding activity after 3 h of EHEC infection in conditions where HCP is produced. No differences in AP-1 DNA binding were observed between suboptimal conditions of HCP production or when the *hcpA* mutant was used. Our data also showed that HCP induced the activation of the NF-κB factor in HT-29 cells. Multiple signaling pathways may converge to activate NF-κB in epithelial cells stimulated by pathogenic *E. coli* [49], [50], *S.* Typhimurium, *Shigella flexneri*, *Helicobacter pylori*, or EPEC [13], [34], [51], [52]. Previous studies showed that the activation of three main members of the MAPK family (ERK1/2, p38, and JNK) is stimulated by the presence of EHEC in Caco-2 and T84 cells [53]. In agreement with these studies, we found that HCP-producing EHEC may stimulate the activation and phosphorylation of p38, ERK1/2 and JNK possibly by IL-8 activation.

The flagellins of *Salmonella* spp. [54]–[56] and other Gram-negative bacteria share similar inflammatory properties suggesting that some degree of sequence or structure conservation which is responsible for inflammatory response induction [57]. The HcpA monomer alone is capable of inducing cytokines release. It remains to be determined what specific sequences of the HcpA protein contribute to the eukaryotic proinflammatory activity demonstrated here. Based on our findings, we conclude that HCP along with LPS and flagella could be responsible for the inflammatory processes undergone during hemorrhagic colitis.

## Materials and Methods

### Bacterial Strains and Growth Conditions

To evaluate the production of HCP EHEC O157:H7 strain EDL933 was cultured in different media [Colonization factor antigen (CFA) agar, Trypticase soy broth (TSB), Luria Bertani (LB) broth, Mueller Hinton (MH), Dulbecco's minimal Eagle medium (DMEM), and Minca minimal medium broth] with shaking, at 37°C and 5% CO_2_. Minca minimal medium contained 1.36 g KH_2_PO_4_, 10.1 g Na_2_HPO_4_.2H_2_O, 1 g glucose, 1 g casaminoacids, 10 g MgSO_4_.7H_2_O, 1 g MnCl_2_.4H_2_O, 0.135 g FeCl_3_.6H_2_O, 0.4 g CaCl_2_.2H_2_O per liter adjusted to pH 7.5 and 1.5% agar [43].

### DNA Manipulations

Restriction enzyme digestion, agarose gel electrophoresis, PCR, plasmid extraction, DNA ligation, bacterial transformation and other routine DNA procedures were performed as described by Sambrook *et al* [58].

### Construction of Isogenic Mutants

Non-polar deletion mutants in *hcpA*, *fliC*, and *hcpA*/*fliC* genes were generated by the lambda Red recombinase method, as previously described [59]. The mutation of the *hcpA* gene in EDL933 was constructed replacing it with a kanamycin-resistance cassette derived from the template plasmid pKD4. The primers used for the mutagenesis of the *hcpA* gene were G68 *hcpA/P1* (5′-AATCAAGGAGCGAAACAGATGGACAAGCAACGCGGTTTTA C ATGTAGGCTGGAGCTGCTTCG-3′) and G69 *hcpA/P2* (5′AATATTCATTGCCGCTCC TTAGTTGGCGTCATCAAAGCGGAACATATGAATATCCTCCTTAG-3′). The *hcpA* mutation was confirmed by selecting colonies grown in LB with kanamycin and by PCR using primers G98 *hcpA-F* (5′TCGCTAGTTGCTGACAGATTT-3′) and G99 *hcpA-R* (5′-AA TGTCTGTTGTGTGCGACTG-3′), as previously described by Xicohtencatl-Cortes *et al* [24]. In addition, a chloramphenicol antibiotic-resistance gene amplified from pKD3 using forward primer G72 (5′-AATATAGGATAACGAATCATGGCACAAGTATTAATACCAA CTGTAGGCTGGAGCTGCTTCG-3′) and reverse primer G73 (5′-TTAATCAGGTTACAA CGATTAACCCTGCAGCAGAGACAGAACCATATGAATATCCTCCTTA-3′) was introduced in the chromosomal *fliC* gene as previously described by Erdem *et al* [60]. Mutants were grown on selective media and the *fliC* mutation was confirmed by PCR using primers G94 (5′-TCCC AGCGATGAAATACTTGC-3′) and G95 (5′-GAGTTATCGGCATG ATTATCC-3′). EDL933 was grown overnight in LB broth at 37°C with shaking, unless otherwise stated. Arabinose was used at a concentration of 100 mM in LB broth to induce λ Red recombinase production from plasmid pKD46 [59]. Antibiotics used in selected media were used at the following final concentrations: kanamycin (100 µg/ml), chloramphenicol, (30 µg/ml), and ampicillin (100 µg/ml).

### Cloning and Expression of His-tagged HcpA

The *hcpA* gene of EDL933 was PCR-amplified with the forward primer hcpA-F (5′-C CGGGATCCTTTACACTTATCGAACTGAT-3′) and reverse primer hcpA-R (5′-GCCAAG CTTTTAGTTGGCGTCATCAAA-3′) and cloned into the pET-28 plasmid (Novagen) yielding a recombinant fusion protein with histidine (6×His) and T7 tags at the amino terminus.

### Antisera

Rabbit anti-PpdD antibody referred to as anti-HCP was a kind gift from O. Francetic (Pasteur Institute) and was used after 6× adsorptions with the EDL933 *hcpA* mutant strain to remove non-specific antibodies [24].

### Immunogold Labeling and Purification of HCP and His-tagged HcpA

For immunoelectron microscopy, bacteria were incubated with anti-HCP serum diluted 1∶10 in PBS containing 1% (w/v) bovine serum albumin (BSA) for 2 h and washed three times with PBS. Goat anti-rabbit IgG labeled with 10-nm gold particles (Sigma-Aldrich) diluted 1∶10 in PBS-BSA was used to detect antibodies bound to the pili and was incubated for 2 h at room temperature. After washing with PBS, the samples were negatively stained with 1% sodium-phosphotungstic acid (pH 7.4) for 5 min and then visualized under a Philips transmission electron microscope (TEM).

For the purification of HCP, EHEC EDL933 was cultured in 50 Minca media plates (150×15 mm) with 1.5% agar, incubated and grown for 24 h, at 37°C and 5% CO_2_. The bacteria were resuspended in 100 mM phosphate buffer, pH 7.4 and the pili were detached by vigorous shaking. The different steps of the HCP purification procedure were monitored by TEM, sodium dodecyl-sulfate polyacrylamide gel electrophoresis (SDS-PAGE) and Coomassie blue staining, as previously described [21]. Briefly, bacteria were separated by centrifugation at 10,000×*g* for 30 min and the supernatant was centrifuged at 18,000×*g* for 30 min to remove flagella, outer membranes and bacterial debris. The pili-containing supernatant was centrifuged at 78,000×*g* for 2 h and the pellet was dissolved in 5 ml phosphate buffer 100 mM and pH 7.4. The suspension containing HCP was applied onto a molecular exclusion chromatography column (Sephadex G-100; Sigma- Aldrich) and the pili were eluted in 0.5 ml fractions with 100 mM sodium phosphate buffer (pH 7.4), and read at OD_280_ nm.

The purification of recombinant HcpA protein was performed according to the manufacturers' procedures (Qiagen). After binding of HcpA-6×His to the nickel column, 20 mM imidazole was used to remove contaminant proteins and the HcpA recombinant protein was eluted with a gradient of 100 to 250 mM of imidazole. The 6×His was removed from HcpA using the procedure described in the Qiagen protocol. HcpA without the 6×His-tag was dissolved in 100 mM phosphate buffer, pH 7.4 and applied onto a molecular exclusion chromatography column (Sephadex G-10; Sigma-Aldrich). Fractions of 0.5 ml were collected and protein content monitored at OD 280 nm. Protein peaks were analyzed by 16% SDS-PAGE stained with Coomassie blue and by Western blotting. To remove LPS (endotoxin), the HCP and HcpA were mixed with polymixin B beads (Sigma-Aldrich) and as previously described [61].

### SDS-PAGE and Western Blot Analyses

HCP and HcpA were routinely separated by SDS-PAGE on 16% acrylamide gels and stained with Coomassie blue. For Western blotting, the proteins were transferred onto a PVDF membrane for 2 h at 90 volts. The immobilized proteins were incubated 1 h with a primary antibody against HCP with a dilution of 1∶3,000 at room temperature. After washing three times with PBS-Tween-20, the membrane was incubated 1 h with goat anti-rabbit IgG conjugated to peroxidase (Sigma-Aldrich). The blots were developed with HyGLO chemiluminescent HRP antibody detection reagent (Amersham) [24]. For the detection of His-tagged HcpA, a mouse monoclonal anti-His antibody (Sigma-Aldrich) was used.

### Quantification of HCP by Flow Cytometry

To determine the production of HCP by flow cytometry, EDL933 strain was cultured in different media [CFA agar, TSB, LB, MH, DMEM, and Minca minimal medium] at 37°C with 5% CO_2_, as previously described [24]. For the assays, 45 µl of each bacterial culture with an optical density of 1.1 were incubated with 40 µl of anti-HCP (1∶2,000) for 2 h. Then, three gentle washes were performed with cold PBS and incubated for 2 h with 40 µl of a 1∶10,000 dilution of goat anti-rabbit IgG (H+L) Alexa Fluor conjugate (Invitrogen, California, USA) and resuspended with 800 ml (final volume) of PBS. Bacteria were labeled with 5 µl of a propidium iodide solution (Sigma-Aldrich) and analyzed in a Becton Dickinson FACS Calibur. The experiments were performed three times by triplicate on separate days and the data were expressed as the mean of the averages.

### Binding of Purified HCP to HT-29 Cells

To determine if HCP have adhesive properties, 50, 100, and 200 µg/ml of purified HCP were applied on the apical surface of polarized HT-29 cells present and incubated for 2 h at room temperature, washed with PBS, and then fixed with 2% formaldehyde. After washing 3 times with PBS, propidium iodide was added to stain the nucleus of the eukaryotic cells and bound HCP was detected with anti-HCP (1∶3,000) serum followed with secondary anti-rabbit Ig antibody conjugated to Alexa-Fluor 488 (1∶10,000). To confirm the results a binding inhibition assay was performed, in which HCP was pre-incubated with a 1∶100 dilution of anti-HCP antibodies for 2 h before its addition to the HT-29 cells. In both cases, the samples were processed and monitored by immunofluorescence microscopy (IFM).

### Induction of Proinflammatory Cytokines in Intestinal and Non-Intestinal Cells

Polarized epithelial cell monolayers (HT-29 and T84) and non-polarized epithelial cells (HeLa) were maintained at 1∶1 mixture of DMEM/Ham's F12 medium (GIBCO BRL)], supplemented with 10% fetal bovine serum (Invitrogen, California, USA), 2 µM L-glutamine, 1.5 g/l sodium bicarbonate, 10 mM Hepes buffer, 0.1 mM non-essential amino acids, 1.0 mM sodium piruvate (90%), 40 µg/ml penicillin and 90 µg/ml streptomycin. Monolayers of intestinal cells were polarized and maintained at 37°C and 5% CO_2_. The monolayers of intestinal and non-intestinal cells were incubated with protein (HCP and HcpA) in the apical surface at various concentrations (0.1, 1, 10, 25, 50, 75, and 100) and at different times (0.5, 1, 2, 4, 6, 12, and 24 h) to evaluate the triggering of cytokines. The culture supernatants were collected from the apical and basolateral surfaces, centrifuged for 10 min at 10,000×*g* (to remove outer membranes and cell debris) and filter sterilized using 0.22-µm-pore-size filters to be analyzed by ELISA [10]. Supernatants obtained from HeLa cells were also filter sterilized using 0.22-µm-pore-size filters. The medium employed for the preparation of epithelial cells and purified flagella from EPEC and EHEC, was used as negative and positive controls, respectively.

The induction of proinflammatory cytokines by the EDL933 O157:H7 and derivative mutants *hcpA*, *fliC* and *hcpA/fliC* was also analyzed by ELISA, after grown in Minca media. Likewise, the wild-type and the *hcpA* mutant were also used to study the induction of proinflammatory cytokines in different media.

### Transepithelial Electrical Resistance Measurements

Colonic intestinal epithelial HT-29 and T84 cells were maintained in the same medium as described above. Polarized epithelial cell monolayers were prepared by seeding 5×10^5^ cells on Transwell-Clear polyester membranes (3-µm-pore-size, 6.5-mm-diam Costar inserts; Costar Corp.) as previously described [10]. Integrity of tight junctions was monitored by transepithelial resistance to passive ion flow with a dual voltage ohmmeter (Millicell-ERS; Millipore Corp.). The polarized T84 and HT-29 cells were not used until the transepithelial resistance of the monolayers reached between 1,500–2,000 Ω/cm^2^ or higher.

### Quantification of Proinflammatory Cytokines by ELISA

A sandwich ELISA using 96-well plates coated overnight at 4°C, and using a concentration of 10 µg/ml goat anti-human antibodies of IL-8 and TNF-α (BD Biosciences Pharmingen, San Diego, CA, USA) was performed as previously described [8]. The supernatants obtained from the apical and basolateral surfaces after 0.5, 1, 4, 6, 12, and 24 h of incubation of HCP with intestinal or non-intestinal cells, were added to the wells followed by rabbit anti-human IL-8 and TNF-α. In different experiments levels of IL-8 and TNF-α were measured in supernatants of intestinal cells incubated with 0.1, 1, 10, 25, 50, 75, and 100 ng/ml of HCP. The complexes were detected with goat anti-rabbit IgG peroxidase conjugate (Sigma-Aldrich, St. Louis, MO, USA) and peroxidase substrate. Color development was read at an optical density of 595 nm.

The detection of the release of other interleukins such as IL-2, IL-4, IL-6 and IL-10 present in the supernatants of the apical and basolateral surfaces, were also measured after 6 h of incubation with 100 ng/ml of purified HCP and the cytokines were detected by ELISA as described above. The IL-2, IL-6, and IL-10 release was detected with monoclonal antibodies (anti-IL-2, anti-IL-6 and IL-10, respectively) (Santa Cruz Biotechnology, Inc.). All determinations of cytokines were performed by triplicate.

### Blocking Cytokines Release in HT-29 Cells

To confirm that HCP and HcpA induce proinflammatory cytokines (IL-8 and TNF-α) production in intestinal cells, HCP or HcpA were incubated at 37°C for 2 h with rabbit polyclonal antibodies against HCP and then were added to the apical surface of polarized HT-29 cells for 6 h. Cytokines were assayed as described above.

### Electrophoretic Mobility Shift Assay (EMSA)

Polarized HT-29 cells were seeded into six-well plates. The cell monolayers were washed and treated with EHEC EDL933 and the *hcpA* mutant in serum- and antibiotic-free DMEM. At the times indicated below, the infected cells were washed with PBS. AP-1 and NF-κB DNA binding activities were analyzed and prepared as described by Dahan *et al* [44]. Oligonucleotide probes contained the consensus sequence for AP-1 site (5′-TTCGTGACTC AGCGG-3′) and for the NF-κB site (5′-ATGTGAGGGGACTTTCCCAGGC-3′). The specificity of the complexes was analyzed by incubation with an excess of unlabeled AP-1 or NF-κB oligonucleotides. Complexes were separated by electrophoresis on a 6% non-denaturing polyacrylamide gel in 0.5× Tris-borate-EDTA buffer. The dried gels were autoradiographed (Amersham Hyperfilms).

HT-29 cells obtained after the infection were washed with PBS and scraped at 4°C with a lysis buffer described by Dahad S. *et al* [44]. The cells were sonicated and solubilized for 30 min at 4°C, and then centrifuged at 14,000×*g* for 20 min at 4°C. The protein concentration of the supernatant was determined by the Bradford method. Equal amounts (50 µg) of whole-cell lysates were subjected to 16% SDS-PAGE gels and the proteins were transferred to a PVDF membrane. After blocking with 5% milk, they were incubated overnight at 4°C with anti-phospho-ERK1/2,anti-phospho-p38, anti-phospho-JNK, anti-phospho-IκB-α, and anti-IκB-α rabbit antibodies (New England Biolabs) or with anti-ERK2, anti-p38, anti-JNK (Santa Cruz Biotechnology, Santa Cruz, Calif.) and horseradish peroxidase-conjugated anti-rabbit antibodies. The presence of antibodies was revealed with the enhanced chemiluminescence detection system.

### Densitometric Analysis

The changes in signal or band intensity were quantified by densitometric analysis using the Quantity One 4.4.1 program (Bio-Rad chemi-doc). All experiments were repeated at least three times and a representative result is shown for each experiment.
